# The Influence of Compositional and Contextual Factors on Non-Receipt of Basic Vaccines among Children of 12-23-Month Old in India: A Multilevel Analysis

**DOI:** 10.1371/journal.pone.0106528

**Published:** 2014-09-11

**Authors:** Daouda Sissoko, Helen Trottier, Denis Malvy, Mira Johri

**Affiliations:** 1 Department of Social and Preventive Medicine, Faculty of Public Health, Université de Montréal, Montreal, Quebec, Canada; 2 Sainte-Justine Hospital Research Center, Montreal, Quebec, Canada; 3 International Health Unit (USI), Centre de Recherche du Centre Hospitalier de l’Université de Montréal (CRCHUM), Montreal, Quebec, Canada; 4 Département des Maladies Infectieuses et Tropicales, Centre Hospitalier Universitaire de Bordeaux, Bordeaux, France; 5 INSERM 897 & Centre René-Labusquière, Université de Bordeaux, Bordeaux, France; 6 Department of Health Administration, School of Public Health, Université de Montréal, Montreal, Quebec, Canada; Brighton and Sussex Medical School, United Kingdom

## Abstract

**Background:**

Children unreached by vaccination are at higher risk of poor health outcomes and India accounts for nearly a quarter of unvaccinated children worldwide. The objective of this study was to investigate compositional and contextual determinants of non-receipt of childhood vaccines in India using multilevel modelling.

**Methods and Findings:**

We studied characteristics of unvaccinated children using the District Level Health and Facility Survey 3, a nationally representative probability sample containing 65 617 children aged 12–23 months from 34 Indian states and territories. We developed four-level Bayesian binomial regression models to examine the determinants of non-vaccination. The analysis considered two outcomes: completely unvaccinated (CUV) children who had not received any of the eight vaccine doses recommended by India’s Universal Immunization Programme, and children who had not received any dose from routine immunisation services (no RI). The no RI category includes CUV children and those who received only polio doses administered via mass campaigns. Overall, 4.83% (95% CI: 4.62–5.06) of children were CUV while 12.01% (11.68–12.35) had received no RI. Individual compositional factors strongly associated with CUV were: non-receipt of tetanus immunisation for mothers during pregnancy (OR = 3.65 [95% CrI: 3.30–4.02]), poorest household wealth index (OR = 2.44 [1.81–3.22] no maternal schooling (OR = 2.43 [1.41–4.05]) and no paternal schooling (OR = 1.83 [1.30–2.48]). In rural settings, the influence of maternal illiteracy disappeared whereas the role of household wealth index was reinforced. Factors associated with no RI were similar to those for CUV, but effect sizes for individual compositional factors were generally larger. Low maternal education was the strongest risk factor associated with no RI in all models. All multilevel models found significant variability at community, district, and state levels net of compositional factors.

**Conclusion:**

Non-vaccination in India is strongly related to compositional characteristics and is geographically distinct. Tailored strategies are required to overcome current barriers to immunisation.

## Introduction

Vaccination is a key strategy for reducing child mortality [1], [2]. In 1974, the World Health Organization (WHO) established the Expanded Programme on Immunization (EPI) to ensure that all children had access to six basic vaccines: Bacille Calmette-Guérin vaccine (BCG), diphtheria-tetanus-pertussis vaccine (DTP), oral poliovirus vaccine (OPV), and measles-containing vaccine (MCV) [3]. A recent report suggests that vaccination against four diseases targeted by the EPI - diphtheria, tetanus, pertussis and measles - averts an estimated 2 to 3 million deaths every year [4], [5]. Despite this success, 22.6 million infants remained unvaccinated (defined as non-receipt of DTP1)-or under-vaccinated (defined as non-receipt of DTP3) worldwide in 2012 [6]. According to the Child Health Epidemiology Reference Group (CHERG), of the estimated 8.8 million deaths of children under 5 years of age worldwide in 2008, 1.5 million (17%) were due to vaccine preventable diseases (VPDs) [7], [8].

Of 12.6 million children who had not received a DTP1 dose in 2012 (commonly considered as proxy for access to vaccination services), approximately 3 million were Indians [6]. Numerous studies have addressed the question of suboptimal childhood vaccination in India. For example, one recent systematic review [9] identified several risk factors related to child (gender, birth order), family (area of residence, wealth, parental education), demography (religion, caste), and community (access to care, community literacy) characteristics. Though the cumulative evidence is impressive, it offers a limited perspective. First, while the characteristics of unvaccinated children are likely to be different from those of undervaccinated children, primary studies have generally considered partially vaccinated and unvaccinated children together, and relatively few have focussed on unvaccinated children [10]. Second, previous studies limited their analysis mainly to individual- and household level-factors using fixed effects models [11], [12]. Third, the few previous researches that examined broader contextual-level factors did not take into account the simultaneous net effects resulting from a wider set of compositional and contextual or community factors [13], [14]. Consequently, an approach restricted to a sole level, either the individual level or the macro-scale of contexts, generates conceptual and practical limiting problems [15], [16].

Considering these limitations, investigation of factors related to non-vaccination is yet to be explored accurately in the context of India. This issue is even more important in regard to the call for universal health care by 2020 in India [17]. Indeed, to scale up coverage successfully contingent to well-known scaling up concern, the problem is not just to reach more children, but to reach those facing specific barriers. It is therefore essential to conduct population-based assessments of patterns, distribution and determinants of non-vaccination in order to identify barriers within subpopulations in every context [18]. Given the complexity of the different relationships between influential variables at individual and contextual levels, it is important to assess their relative contribution in a multilevel model that can properly account for individual and contextual factors and their potential interactions.

Our objective was to investigate the role of potential compositional and contextual determinants of non-receipt of basic vaccines among 12–23-month-old children in India. Specifically, we aimed to: (i) ascertain whether individual or household level (compositional) factors are significantly associated with childhood non-vaccination, net of community-level factors in India; (ii) determine whether there was a significant contextual variation of childhood non-vaccination; (iii) assess whether contextual variation was explained by individual- and contextual-level factors.

## Methods

We used data from the District Level Household and Facility Survey 3 (DLHS-3), a nationwide household survey at district level, conducted in 2007–2008 in 34 Indian states and territories [19]. The DLHS-3 was designed as a cross-sectional study that used a stratified, systematic, multistage cluster sampling design [19].

### Outcome measure

The basic Indian vaccination schedule is proposed by Universal Immunization Programme (UIP). The UIP is the largest immunization program in the world and targets 27 million infants annually. The UIP protects children against 7 vaccine-preventable diseases: tuberculosis, diphtheria, tetanus, pertussis, polio, measles (added in 1985) and hepatitis (added in 1990). Vaccines are provided free of cost and delivered through strategies such as routine immunization, village health and nutrition days, and outreach campaigns [20]. In keeping with the definition in standard use in India, full immunisation is defined as a child 12–23 months of age receiving all of the following vaccines: a dose of BCG vaccine at birth (or as soon as possible); three doses of DPT vaccine at 6, 10 and 14 weeks of age; at least three doses of OPV at 6, 10 and 14 weeks of age; and one dose MCV at 9 months of age. Vaccination information of 12–23 month-old children in DLHS-3 was obtained either from health cards or from mother’s or caregiver verbal reports.

We created two binary outcomes to study non-vaccination in this sample. First, children 12–23 months of age who had not received any of the following eight vaccine doses (1 dose of BCG vaccine, 3 doses each of DTP vaccine and OPV, and 1 dose of MCV were considered completely unvaccinated (CUV), and were compared to children who had received at least one dose of vaccine. Second, children were considered to have received no routine immunisation (no RI) if they had not received any of the five recommended doses administered only through routine services (1 dose of BCG vaccine, 3 doses of DTP vaccine, and 1 dose of MCV), and were compared to children who had received at least one routine immunisation dose. Full immunization coverage can be attained only through improving routine immunisation systems. For several decades, as part of the global eradication initiative, India has had a very strong polio programme operating largely in campaign mode in parallel to routine immunization services [21], [22]. We therefore also studied those children 12–23 months of age who had not received a single dose of vaccine from routine immunization services.

### Explanatory variables

#### Individual and household (compositional) characteristics

We included the following compositional variables: child sex (male or female), birth order (1, 2, 3, 4 and more), mother’s age (15–24, 25–34, or 35 years or older), mother’s and father’s educational attainment (0 year, 1–5 years, 6–8 years, 9–10 years, 11–12 years, or 13 or more years), caste (scheduled tribe, scheduled caste, other backward caste -OBC- and general), religion (Hindu, Muslim and others i.e. Sikh, Christian, Buddhist and others), antenatal care –ANC- (prenatal visits, tetanus injection during pregnancy), postnatal care (No PNC within 2 weeks), and household wealth. Household wealth index was computed by combining household assets and material possessions by IIPS and divided into quintiles (poorest to the richest groups accounting for the lowest to the highest quintiles).

#### Contextual characteristics

Contextual characteristics are defined at community, district and state levels. State-level characteristics considered included area of residence (urban and rural) and region of residence categorised into two groups as follows:

- The first group included Empowered Action Group States (EAG) and Assam (EAGA). The EAG states, which account for about 45% of India’s population and have particularly high fertility and mortality indicators, were designated as “High Focus States” by the Indian Government in 2001. Due to lagging social and demographic indicators, Assam is often considered with this group. EAGA states were: Assam, Bihar, Chhattisgarh, Jammu and Kashmir, Jharkhand, Madhya Pradesh, Orissa, Rajasthan, Uttar Pradesh, Uttarakhand).
- The second group (other states) included: Arunachal Pradesh, Manipur, Meghalaya, Mizoram, Sikkim, Tripura, Andaman and Nicobar Islands, Andhra Pradesh, Chandigarh, Dadra and Nagar Haveli, Daman and Diu, Delhi, Goa, Gujarat, Haryana, Himachal, Pradesh, Karnataka, Kerala, Lakshadweep, Maharashtra, Pondicherry, Punjab, Tamil Nadu, West Bengal).

We used the term community to describe clustering within the same geographical living environment. Communities were based on sharing a common primary sample unit (PSU) within the DLHS-3 data as it is the most consistent measure of community in the DHS surveys [23]. Since poverty and education characteristics of communities were not directly available, they were constructed by aggregating individual-level characteristics at the PSU level. Specifically, these weighted measures were derived by summing the values obtained on individual women in each community and dividing then by the total number of women respondents living in each one. The community’s poverty status was defined as the proportion of households below 20% of wealth index. The proportion of women with no formal education was generated from native individuals in the database and aimed to represent female illiteracy in the community. In our study, these group-measures were based on an average of 3 women per community (from 1 to 31), which provides a sufficient number 1) to generate reliable estimates [24] and 2) to use Monte Carlo Markov Chains for achieving our computations [25].

### Statistical analysis

The entire national sample (n = 65,617) of children aged 12–23 months was analyzed. Data typically have a hierarchical structure in which children were nested within mothers, mothers were clustered within households, households were nested within communities which were clustered within districts, and finally districts were nested within states. To account for unequal selection probabilities and ensure representativeness of the sample, we applied the appropriate sampling weights.

Determinants of non-vaccination were assessed by using Bayesian binomial regression models. We specified a 4-level model for each binary outcome *y, i.e.,* non-vaccination, for child *i* living in community *j* in district *k* and state *l*. Probability was related to a set of categorical predictors *X* and a random effect for each level by a logit-link function as logit (π_ijkl_) = β_0_+βX+u_0jkl_+v_0kl+_f_0l_. A child level was defined by collapsing child-, mother- and household-level data. The linear predictor of the equation consisted of a fixed part (β0+βX) estimating the conditional coefficients for the covariates. The 3 random intercepts were respectively attributable to communities (u_0jkl_), districts (v_0kl_) and states (f_0l_), each assumed to have an independent and identical distribution and variance estimated at a corresponding level. All models were estimated by using Bayesian methods implemented via Markov Chain Monte Carlo (MCMC) simulation and the Metropolis-Hastings algorithm [26]. We used diffuse default prior distribution for all parameters [26]. Starting values of the distribution were derived from two previous estimations using Iterated Generalized Least Squares (*IGLS)* and second order penalised quasi-likelihood linearization (PQL2). MCMC estimation was adopted in the analysis to reduce bias in the estimates of random effect parameters. Indeed, such bias can arise when multilevel models with discrete outcomes are estimated using maximum-likelihood procedures [27]–[29].

All estimations were performed by MLwiN within STATA 12 MP (Stata, Corp.) and MLWiN 2.26 through runmlwin procedure [30]. Our computations were based on chains of length 50 000 iterations after a burn-in of 5000. Bayesian deviance information criterion (BIC) was used to estimate the goodness of fit of consecutive models [31] The BIC values for each model were compared, and the model with the lowest value was considered the better one for hierarchical models [31].

We examined separately the association between non-vaccination and compositional (individual-household) and contextual variables. The first model is a null model (Model 1), which provides information on the extent to which communities, districts and states vary and further justify assessing random effects at these levels. Model 2 included only individual characteristics while model 3 contained community characteristics. Model 4 expanded model 3 by adding individual level variables. We further fitted a fifth model to analyse a cross-level interaction between household wealth and area of residence (rural and urban). Since we found a significant (p = 0.035) interaction term (area of residence * wealth index), we present separate models including all individual and contextual variables stratified for rural and urban areas of residence.

The fixed effects, i.e., the association between non-vaccination and selected variables, were shown as odds ratio (OR) with its 95% credible interval (CrI). Meanwhile, random effects (measures of variation) were estimated by median odds ratio (MOR) rather than using intra-cluster correlation (ICC) which is better fitted for linear models [32], [33]. The MOR quantifies the unexplained contextual heterogeneity, otherwise it quantifies contextual-level variance on the odds ratio scale and is always greater than or equal to 1 [32].

### Ethical considerations

This study is based on an analysis of existing survey data with all identifier information removed. The survey was approved by the Ministry of Health & Family Welfare, Government of India and the International Institute of Population Sciences (IIPS) institutional review board. All study participants gave informed consent before participation and all information was collected confidentially. Data of DLHS-3 were obtained from the IIPS as they are made available in the public domain for analysis by researchers. Therefore, no additional ethics review is required for this work by the Montréal University committee of ethics.

## Results

**Figure 1:**
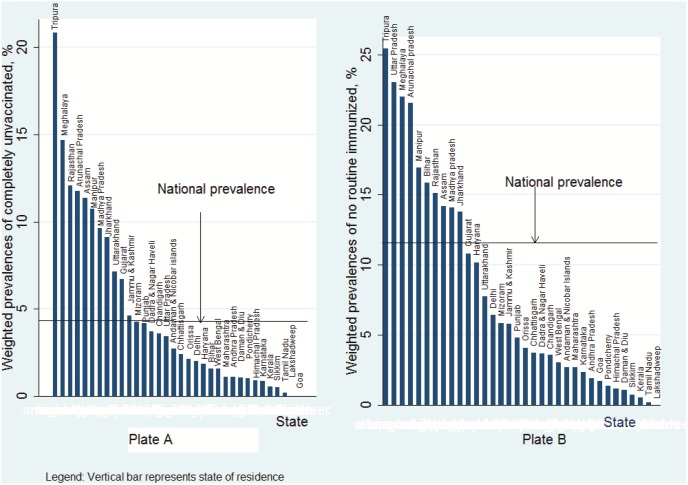
Weighted prevalences of completely unvaccinated and no routine immunized by state, India DLHS-3, 2007–2008.

Of 65 617 children aged from 12 to 23 months, 3173 (4.83%, [95% Confidence Intervals (CI): 4.62–5.06]) were completely unvaccinated (CUV) and failed to receive any of the eight recommended vaccine doses while 7883 (12.01%, [95% CI: 11.68–12.35]) did not receive any vaccine dose through routine services (No-RI). The distribution of CUV and No-RI children showed substantial variation between states. The weighted prevalence of CUV extended from 0% (Goa and Lakshadweep) to 20.9% (Tripura) (Figure 1 Plate A) while that of No-RI ranged from 0% (Lakshadweep) to 25.4% (Tripura) (Figure 1 Plate B). Proportions of non-receipt of basic vaccines varied according to characteristics of children, parents and households (Tables 1 & 2).

**Table 1:** Weighted proportions of non-vaccination by individual characteristics among 65617 children aged 12–23 months, India, DLHS-3, 2007–2008.

Characteristics	Completely Unvaccinated(CUV)	No Routine Immunisation(No RI)
Individual-related characteristics	Number (weighted %)	Number (weighted %)
**Child (n = 65617)**		
**Sex**	3189 (5.0)	7921 (12.0)
Male	1620 (4.8)	3945 (11.0)
Female	1569 (5.0)	3975 (13.0)
**Birth order (n = 64637)**	3155 (5.0)	7796 (12.0)
1	901 (4.0)	1771 (8.0)
2	972 (4.0)	1540 (9.0)
3	509 (5.0)	1274 (13.0)
≥4	1073 (7.0)	3211 (22.0)
**Parents (n = 65617)**		
**Mother’s schooling, years**	3189 (5.0)	7921 (12.0)
0	2225 (8.0)	5976 (20.0)
1–5	451 (5.0)	991 (10.0)
6–8	309 (3.0)	577 (6.0)
9–12	183 (1.0)	352 (3.0)
≥13	21 (1.0)	25 (1.0)
**Mother’s marital status (n = 65617)**	3189 (5.0)	7921 (12.0)
Currently married	3138 (5.0)	7816 (12.0)
Currently alone (single, divorced, widowed, deserted)	51 (7.0)	105 (15.0)
**Mother’s age group, years (n = 65617)**	3189 (5.0)	7921 (12.0)
15–19	303 (5.0)	587 (13.0)
20–24	1075 (4.0)	2570 (10.0)
25–34	1567 (5.0)	3801 (12.0)
35–49	344 (7.0)	963 (20.0)
**Mother received four ANC during pregnancy (n = 64701)**	3158 (5.0)	7803 (12.0)
Yes	88 (1.0)	166 (2.0)
No	3070(5.7)	7637 (14.0)
**Tetanus toxin injection during pregnancy (n = 64701)**	3159 (5.0)	7803 (12.0)
Yes	873 (2.0)	2541 (5.0)
No	2286 (12.0)	5262 (29.0)
**Postnatal care within 2 weeks (n = 61613)**	3121 (5.0)	7715 (12.0)
Yes (reference)	525 (2.0)	1555 (6.0)
No	2595 (8.0)	6160 (18.0)
**Husband’s education, years**		
0	3189 (5.0)	7921 (12.0)
1–5	1368 (9.0)	3557 (22.0)
6–8	595 (6.0)	1506 (14.0)
9–12	554 (5.0)	1237 (11.0)
≥13	578 (3.0)	1414 (7.0)

**Table 2:** Weighted proportions of non-vaccination by household and contextual characteristics among 65617 children aged 12–23 months, India, DLHS-3, 2007–2008.

	Completely Unvaccinated (CUV)	No Routine Immunisation (No-RI)
Characteristics	Number (weighted %)	Number (weighted %)
**Caste group (n = 64424)**	3084 (5.0)	7780 (12.0)
Scheduled caste	575 (5.0)	1553 (12.0)
Scheduled tribe	1059 (10.0)	1609 (15.0)
Other backward caste (OBC)	1003 (4.0)	3513 (13.0)
General	447 (3.0)	1104 (8.0)
**Religion (n = 65614)**	3189 (5.0)	7920 (12.0)
Hindu	2131 (4.0)	5366 (11.0)
Muslims	602 (6.0)	1868 (19.0)
Others/no religion	451 (7.0)	686 (11.0)
**Household-related characteristics**		
***Wealth index*** ** (n = 65603)**	**3189 (5.0)**	**7921 (12.0)**
Poorest	1145. (9.0)	2853 (21.0)
Poorer	919 (7.0)	2265 (16.0)
Middle	630 (5.0)	1483 (11.0)
Richer	362 (3.0)	950 (7.0)
Richest	133 (1.0)	370 (3.0)
**Contextual-related characteristics**		
***Place of residence (n = 65617)***	**3189 (5.0)**	**7921 (12.0)**
Rural	2820 (5.0)	6931 (13.0)
Urban	369 (3.0)	990 (8.0)
**Region ** ***(n = 65617)***	**3189 (5.0)**	**7921 (12.0)**
EAG-Assam	2276 (6.0)	6322 (16.0)
Other	913 (3.0)	1599 (6.0)

CUV: received none of the eight basic vaccine doses; No-RI: received none of the five recommended vaccine doses delivered exclusively through the routine immunisation system; EAG: Empowered- Action Group; wealth index based on household amenities and possessions calculated by International Institute for Population Science.

Tables 3&4 present four-level univariate logistic regression results. Increasing maternal and paternal educational attainment was protective against child non-receipt of vaccines through a graded trend (P trend <0.0001) (table 3). Children at birth ranks ≥3 were more likely to be unvaccinated. Lack of utilisation of health services during and after pregnancy by the mother was positively associated to being CUV. In comparison to the reference group, children born from women with less than 4 ANC visits or no tetanus injection during pregnancy had nearly 8 time the risk of being CUV (OR = 7.56 [95% Credible Interval (CrI): 6.09–9.37] and OR = 7.25 [6.69–7.85], respectively). Furthermore, not receiving PNC within 2 weeks after birth was also associated with CUV status (OR = 4.16 [3.78–4.57]). Finally, when contrasting both outcomes, the strength of association of variables related to individual characteristics appeared generally more pronounced for No-RI while magnitudes were larger among CUV for variables related to health services utilization by mother and household wealth index.

**Table 3:** Four-level univariate logistic regression modeling of individual factors associated with non-vaccination among children aged 12–23 months, India, 2007–2008.

Characteristics	Completely Unvaccinated (CUV)		No Routine Immunisation (No-RI)	
**Weighted Prevalence, % [95% CI]**	4.83 (4.62–5.06)		12.01 (11.68–12.35)	
	**Odds Ratio (95% CrI)**	**P-value**	**Odds Ratio (95% CrI)**	**P-value**
**Individual-related characteristics**				
**Child**				
**Sex** Male (Reference)	1	0.145	1	0.003
Female	1.05 (0.98–1.13)		1.13 (1.08–1.20)	
**Birth order** 1 (Reference)	1	<0.001	1	<0.0001
2	0.97 (0.87–1.07)		1.16 (1.08–1.25)	
3	1.25 (1.11–1.40)		1.44 (1.32–1.57)	
≥4	1.91 (1.75–2.10)		2.16 (2.00–2.33)	
**Parents**				
**Mother’s schooling years**		<0.0001		<0.0001
0	12.50 (8.12–19.24)		19.56 (12.87–29.75)	
1–5	7.86 (5.07–12.20)		10.30 (6.74–15.74)	
6–8	4.95 (3.18–7.72)		6.10 (3.97–9.33)	
9–12	2.13 (1.35–3.35)		2.95 (1.91–4.55)	
≥13 (Reference)	1		1	
**Currently alone versus currently married**	1.66 (1.25–2.19)	<0.0001	1.42 (1.11–1.82)	<0.0001
**Mother’s age group, years**		<0.0001		<0.0001
15–19 (Reference)	1		1	
20–24	0.92 (0.79–1.08)		0.81 (0.72–0.90)	
25–34	1.17 (1.00–1.37)		0.99 (0.89–1.10)	
35–49	1.68 (1.40–2.01)		1.39 (1.22–1.58)	
**Less than 4 ANC visits**	7.56 (6.09–9.37)	<0.0001	4.11 (3.82–4.43)	<0.0001
**No Tetanus toxin injection during pregnancy**	7.25 (6.69–7.85)	<0.0001	5.60 (5.23–6.00)	<0.0001
**No PNC visit within 2 weeks**	4.16 (3.78–4.57)		2.71 (2.52–2.92)	<0.0001

**ANC** Antenatal consultation; **PNC** Postnatal consultation; 95% CI: 95% Confidence interval.

**Table 4:** Four-level univariate logistic regression modeling of household and contextual factors associated with non-vaccination among children aged 12–23 months, India, 2007–2008.

Characteristics	Completely Unvaccinated (CUV)		No Routine Immunisation (No-RI)	
	Odds Ratio (95% CI)	P-value	Odds Ratio (95% CI)	P-value
**Husband’s Educational attainment, y**		<0.0001		<0.0001
0	6.58 (5.29–8.20)		6.87 (5.84–8.07)	
1–5	4.46 (3.55–5.60)		4.67 (3.95–5.51)	
6–8 years	3.60 (2.86–4.53)		3.43 (2.90–4.05)	
9–12 years	2.01 (1.60–2.53)		2.10 (1.79–2.47)	
≥13 years (reference)	1		1	
**Caste group General (reference)**	1	<0.0001	1	<0.0001
Other backward caste (OBC)	1.19 (1.06–1.33)		1.41 (1.30–1.54)	
Scheduled caste	1.43 (1.26–1.63)		1.60 (1.46–1.76)	
Scheduled tribe	3.29 (2.94–3.69)		2.45 (2.17–2.77)	
**Religion** Hindu (reference)	1	<0.0001	1	<0.0001
Muslims	1.49 (1.36–1.64)		1.96 (1.80–2.13)	
Others/no religion	1.87 (1.68–2.07)		1.23 (1.03–1.46)	
**Household’s** Wealth index		<0.0001		<0.0001
Poorest	8.29 (6.88–9.99)		7.82 (6.78–9.03)	
Poorer	6.21 (5.14–7.50)		5.40 (4.69–5.22)	
Middle	4.48 (3.69–5.44)		3.69 (3.19–4.23)	
Richer	2.48 (2.02–3.04)		2.37 (2.05–2.74)	
Richest (reference)	1		1	
**Contextual-level characteristics**				
Rural versus urban residence	1.82 (1.63–2.04)	<0.0001	1.69 (1.51–1.87)	<0.0001
EAG-Assam states versus other states	1.08 (0.46–2.07)	0.117	1.95 (0.87–4.34)	0.132
Community Illiterate women	1.12 (0.89–1.40)	0.886	0.91 (0.80–1.02)	0.511
Community poverty	0.89 (0.58–1.31)	0.220	1.05 (0.90–1.22)	0.107

EAG: Empowered- Action Group; 95% CI: 95% Confidence interval.

Multivariate four-level regression results adjusted for potentially confounders showed results of association between CUV and individual (Table 5) and contextual-level (Table 6) variables. Model 2 shows associations for individual-level factors. Firstly, children born from mothers with no schooling relative to those born from mothers having at least 13 years of schooling were almost two and a half times more likely to be CUV. A similar but less marked trend was observed with respect to father’s educational attainment. Secondly, variables related to health services utilization by the mother remained significant, particularly maternal tetanus immunization during pregnancy which was the strongest individual-level factor related to CUV status. Thirdly, the association between CUV and wealth index showed a significant, dose-response relationship indicating that the risk of being CUV increased with lower household wealth. The effects of the inclusion of contextual factors are shown in Table 6 (Model 3). Rural residence and living in an EAG-Assam state increased the likelihood of a child being CUV with respective ORs of 1.57 [1.33–1.81] and 3.15 [1.14–6.86]. In Model 4 (Table 6), the inclusion of the community-level variables had minimal effect on the contribution of compositional variables on the likelihood of being CUV. Inversely, the effect of living in rural settings reversed and became protective against being CUV (OR = 0.71 [0.59–0.86]) while the effect of living in an EAG-Assam state disappeared.

**Table 5:** Four-level multivariate logistic regression modeling of fixed and random-effect of individual factors associated to non-vaccination (completely unvaccinated, CUV) among 12–23 months children in India, 2007–2008.

	Model 1 (Empty) OR(95% CrI)	Model 2 (Individual) OR(95% CrI)	Model 3 (Contextual) OR(95% CrI)	Model 4 (Individual & Contextual)OR (95% CrI)
**Individual-level factors**				
Sex Female vs. male		1.09 (1.00–1.20)		1.09 (1.00–1.19)
Birth order 1 (reference)		1		1
2		0.93 (0.82–1.08)		0.93 (0.81–1.06)
3		0.97 (0.82–1.13)		0.97 (0.82–1.13)
≥4		1.10 (0.94–1.28)		1.10 (0.94–1.27)
**Mother’s schooling, years**				
0		2.41 (1.31–3.96)		2.43 (1.41–4.05)
1–5		1.80 (1.01–2.95)		1.82 (1.04–3.03)
6–8		1.68 (0.95–2.79)		1.69 (0.97–2.11)
9–12		1.01 (0.57–1.67)		1.02 (0.59–1.69)
≥13 (reference)		1		1
**Mother’s age group, years**				
15–19 (reference)		1		1
20–24		0.98 (0.78–1.20)		0.98 (0.80–1.19)
25–34		1.04 (0.82–1.29)		1.04 (0.83–1.28)
35–49		0.92 (0.70–1.19)		0.92 (0.71–1.19)
**Less than 4 ANC visits**		1.52 (1.12–1.96)		1.53 (1.25–1.88)
**No TTI during pregnancy**		4.25 (3.78–4.78)		3.65 (3.30–4.02)
**No PNC within 2 weeks**		1.89 (1.61–2.12)		1.81 (1.59–2.04)
**Father’s schooling, years**				
0		1.81 (1.32–2.40)		1.83 (1.30–2.48)
1–5		1.43 (1.03–1.91)		1.44 (1.03–1.97)
6–8		1.41 (1.03–1.88)		1.43 (1.04–1.94)
9–12		1.14 (0.85–1.51)		1.17 (0.85–1.57)
≥13 (reference)		1		1

**ANC** Antenatal consultation; **PNC** Postnatal consultation; TTI: Tetanus Toxin Injection; 95% CI: 95% Confidence interval.

**Table 6:** Four-level multivariate logistic regression modeling of fixed and random-effect of individual and contextual factors associated to non-vaccination (completely unvaccinated, CUV) among 12–23 months children in India, 2007–2008.

	Model 1 (Empty) OR (95% CrI)	Model 2 (Individual) OR (95% CrI)	Model 3 (Contextual) OR (95% CrI)	Model 4 (Individual & Contextual) OR(95% CrI)
**Caste group** General (reference)		1		1
Other backward caste (OBC)		1.04 (0.88–1.21)		1.03 (0.87–1.20)
Scheduled caste		1.19 (1.00–1.42)		1.17 (1.00–1.40)
Scheduled tribe		1.46 (1.18–1.78)		1.46 (1.19–1.78)
**Religion** Hindu (reference)		1		1
Muslims		1.89 (1.57–2.24)		1.82 (1.52–2.16)
Others/no religion		1.04 (0.80–1.32)		1.05 (0.81–1.33)
**Household Wealth index**				
Poorest		2.10 (1.58–2.76)		2.44 (1.81–3.22)
Poorer		1.78 (1.36–2.31)		2.05 (1.57–2.49)
Middle		1.59 (1.22–2.05)		1.80 (1.36–2.32)
Richer		1.36 (1.05–1.75)		1.48 (1.30–1.91)
Richest (reference)		1		1
**Contextual-level factors**				
Rural vs. Urban			1.57 (1.33–1.81)	0.71 (0.59–0.86)
EAG-Assam versus other			3.15 (1.14–6.86)	1.09 (0.49–2.47)
Community Illiterate women			1.05 (0.84–1.32)	1.09 (0.86–1.37)
Community poverty			0.93 (0.62–1.34)	0.90 (0.58–1.32)
Variance (SE)				
Community	2.04 (0.13)	1.66 (0.12)	2.02 (0.12)	1.69 (0.13)
District	1.15 (0.12)	0.84 (0.10)	1.14 (0.12)	0.83 (0.10)
State	1.93 (0.60)	1.06 (0.35)	1.72 (0.54)	1.10 (0.37)
MOR				
Community	3.90 (3.58–4.25)	3.42 (3.12–3.73)	3.88 (3.59–4.20)	3.44 (3.14–3.79)
District	2.78 (2.51–3.09)	2.40 (2.18–2.65)	2.77 (2.50–3.08)	2.39 (2.17–2.64)
State	3.76 (2.66–5.77)	2.67 (2.03–3.75)	3.79 (2.51–5.27)	2.72 (2.04–3.44)

EAG: Empowered- Action Group; **MOR:** Median odds ratio; **95% CrI**: Credible interval.

Tables 7&8 presents multivariate logistic regression results related to non-receipt of routine immunisation. As compared to CUV, being No-RI (Model 4, Tables 7&8) was more strongly associated to mother’s educational attainment, being Muslim, female gender, and living in urban areas. Notably, maternal education was the strongest factor. Other findings were similar to those in Tables 5&6.

**Table 7:** Four-level multivariate logistic regression modeling of fixed and random-effect of individual actors associated with non-receipt of routine immunisation (No-RI) among 12–23 months children in India, 2007–2008.

	Model 1 (Empty) OR (95% CrI)	Model 2 (Individual) OR (95% CrI)	Model 3 (Contextual) OR (95% CrI)	Model 4 (Individual & Contextual) OR (95% CrI)
**Individual-level factors**				
Sex Female vs. male		1.18 (1.11–1.25)		1.18 (1.11–1.25)
Birth order 1 (reference)		1		1
2		1.09 (0.98–1.19)		1.08 (0.99–1.19)
3		1.21 (1.09–1.35)		1.20 (1.08–1.35)
≥4		1.44 (1.29–1.60)		1.42 (1.28–1.59)
**Mother’s schooling, years**				
0		4.35 (3.82–6.18)		4.92 (3.18–6.94)
1–5		3.13 (2.03–4.50)		3.55 (2.29–4.99)
6–8		2.42 (1.58–3.47)		2.73 (1.75–3.82)
9–12		1.77 (1.16–2.54)		1.99 (1.29–2.78)
≥13 (reference)		1		1
**Mother’s age group, years**				
15–19 (reference)		1		1
20–24		0.80 (0.70–0.91)		0.80 (0.70–0.91)
25–34		0.74 (0.64–0.85)		0.74 (0.64–0.84)
35–49		0.71 (0.60–0.84)		0.70 (0.59–0.84)
**Less than 4 ANC visits**		1.73 (1.45–2.07)		1.75 (1.42–2.06)
**No TTI during pregnancy**		4.00 (3.73–4.30)		4.02 (3.73–4.33)
**No PNC within 2 weeks**		1.55 (1.43–1.67)		1.56 (1.43–1.69)
**Father’s schooling, years**				
0		2.02 (1.65–2.45)		1.93 (1.63–2.35)
1–5		1.58 (1.35–2.05)		1.60 (1.35–1.96)
6–8		1.53 (1.25–1.86)		1.47 (1.22–1.79)
9–12		1.30 (1.07–1.57)		1.27 (1.07–1.53)
≥13 (reference)		1		1
**Caste group General** (reference)		1		1
Other backward caste (OBC)		1.04 (0.88–1.21)		1.10 (0.99–1.22)
Scheduled caste		1.17 (1.04–1.31)		1.15 (1.01–1.30)
Scheduled tribe		1.40 (1.21–1.77)		1.41 (1.21–1.63)

**ANC;** Antenatal care; **PNC;** Postnatal care; **TTI:** tetanus Toxin injection; **95% CrI**: Credible interval.

**Table 8:** Four-level multivariate logistic regression modeling of fixed and random-effect of individual and contextual actors associated with non-receipt of routine immunisation (No-RI) among 12–23 months children in India, 2007–2008.

	Model 1 (Empty)	Model 2 (Individual)	Model 3 (Contextual)	Model 4 (Individual & Contextual)
**Religion** Hindu (reference)		1		1
Muslims		2.10 (1.89–2.33)		2.00 (1.81–2.22)
Others/no religion		1.09 (0.89–1.34)		1.13 (0.92–1.33)
**Household Wealth index**				
Poorest		1.84 (1.53–2.19)		2.27 (1.73–2.73)
Poorer		1.55 (1.29–1.83)		1.89 (1.59–2.27)
Middle		1.33 (1.12–1.57)		1.58 (1.32–1.88)
Richer		1.24 (1.05–1.45)		1.39 (1.17–1.64)
Richest (reference)		1		1
**Contextual-level factors**				
Rural vs. Urban			1.27 (1.14–1.41)	0.61 (0.54–0.68)
EAG-Assam states versus other states			5.96 (2.12–15.04)	1.91 (0.77–6.73)
Community Illiterate women			0.88 (0.73–1.07)	1.00 (0.99–1.00)
Community Poverty			1.02 (0.50–1.86)	0.84 (0.62–1.11)
**Variance (SE)**				
Community	1.36 (0.07)	1.03 (0.07)	1.36 (0.07)	1.01 (0.06)
District	0.69 (0.06)	0.37 (0.04)	0.68 (0.06)	0.37 (0.04)
State	2.10 (0.63)	0.97 (0.32)	1.76 (0.54)	0.99 (0.36)
**MOR**				
Community	3.04 (2.89–3.21)	2.63 (2.46–2.80)	3.05 (2.89–3.22)	2.61 (2.47–2.77)
District	2.21 (2.06–2.39)	1.79 (1.68–1.90)	2.20 (2.05–3.37)	1.79 (1.69–1.91)
State	3.98 (2.83–6.11)	2.56 (1.99–3.53)	3.54 (2.56–5.32)	2.58 (1.77–3.72)

EAG: Empowered- Action Group; MOR: Median odds ratio; 95% CrI: Credible interval.

Tables 9&10 presents results stratified by area of residence (rural versus urban) for CUV and no-RI. Maternal education attainment continued to influence the risk of being CUV uniquely among urban children. Conversely, father’s schooling remained significant in urban and rural areas although its magnitude was larger in urban areas. Interestingly, the household wealth index had almost no effect on risk of being CUV in urban areas but remained a strong determinant of CUV in rural settings. Maternal education remained the strongest determinant of No-RI in both settings whilst the influence of higher birth rank appeared more marked as well as being a girl in urban area.

**Table 9:** Four-level multivariate logistic regression modeling of individual factors associated with non-vaccination among 12–23 months children by area of residence in India, 2007–2008 (model 5).

	Rural CUV	Rural No-RI	Urban CUV	Urban No-RI
** Sex** Female vs. male	1.05 (0.95–1.15)	1.16 (1.09–1.23)	1.26 (1.01–1.61)	1.34 (1.13–1.59)
** Birth order** 1 (reference)	1	1	1	1
2	0.93 (0.80–1.07)	1.07 (0.96–1.18)	0.93 (0.64–1.27)	1.22 (0.94–1.57)
3	0.94 (0.79–1.10)	1.14 (1.01–1.27)	1.08 (0.71–1.53)	1.88 (1.40–2.51)
≥4	1.13 (0.95–1.32)	1.40 (1.26–1.55)	0.88 (0.56–1.27)	1.83 (1.34–2.47)
**Mother’s schooling, years**				
0	1.09 (0.92–1.83)	4.55 (2.55–6.33)	3.39 (1.43–7.88)	7.39 (4.10–13.84)
1–5	0.88 (0.57–1.42)	3.45 (1.89–4.82)	1.60 (0.65–3.79)	3.73 (1.95–7.34)
6–8	0.77 (0.49–1.24)	2.47 (1.36–3.47)	2.29 (0.98–5.16)	4.06 (2.22–7.53)
9–12	0.48 (0.30–1.78)	1.96 (1.06–2.81)	1.25 (0.53–2.79)	2.30 (1.25–4.18)
≥13 (reference)	1	1	1	1
**Mother’s age group, years**				
15–19 (reference)	1	1	1	1
20–24	0.93 (0.75–1.13)	0.81 (0.69–0.91)	1.87 (1.01–3.35)	0.75 (0.52–1.03)
25–34	0.98 (0.77–1.18)	0.75 (0.63–0.86)	2.03 (1.04–3.66)	0.67 (0.44–0.92)
35–49	0.88 (0.67–1.15)	0.72 (0.59–0.85)	1.81 (0.80–3.55)	0.63 (0.38–0.97)
**Less than 4 ANC visits**	1.65 (1.08–2.77)	1.66 (1.32–2.01)	1.31 (0.78–2.07)	1.85 (1.42–2.35)
**No TTI during pregnancy**	4.33 (3.78–4.91)	4.05 (3.76–4.36)	3.95 (3.06–5.03)	3.83 (3.14–4.64)
**No PNC within 2 weeks**	1.75 (1.49–2.00)	1.59 (1.46–1.73)	1.91 (1.40–2.59)	1.58 (1.26–1.87)
**Father’s schooling, years**				
0	1.85 (1.36–2.52)	1.83 (1.49–2.48)	2.20 (1.15–3.91)	2.56 (1.54–4.48)
1–5	1.47 (1.09–1.98)	1.54 (1.25–1.94)	1.79 (1.01–3.16)	1.93 (1.12–3.35)
6–8	1.44 (1.06–1.96)	1.41 (1.15–1.77)	1.90 (1.01–3.21)	1.75 (1.05–3.03)
9–12	1.20 (0.87–1.61)	1.23 (1.01–1.53)	1.40 (0.78–2.31)	1.35 (0.83–2.24)
≥13 (reference)	1	1	1	1

CUV: received none of the eight basic vaccine doses; No-RI: received none of the five recommended vaccine doses delivered exclusively through the routine immunisation system; ANC: Antenatal care; **PNC:** Postnatal care; **TTI:** tetanus Toxin injection; **95% CrI:** Credible interval.

**Table 10:** Four-level multivariate logistic regression modeling of individual and contextual factors associated with non-vaccination among 12–23 months children by area of residence in India, 2007–2008 (model 5).

	Rural CUV	Rural No-RI	Urban CUV	Urban No-RI
**Caste group** General (reference)	1	1	1	1
Other backward caste (OBC)	1.10 (0.89–1.31)	1.10 (1.00–1.22)	0.79 (0.55–1.12)	1.09 (0.85–1.37)
Scheduled caste	1.20 (0.96–1.46)	1.13 (1.01–1.28)	1.12 (0.75–1.64)	1.25 (0.93–1.69)
Scheduled tribe	1.50 (1.17–1.86)	1.39 (1.20–1.61)	1.38 (0.77–2.38)	1.28 (0.79–2.00)
**Religion** Hindu (reference)	1	1	1	1
Muslims	1.88 (1.52–2.31)	2.01 (1.78–2.26)	1.74 (1.26–2.36)	1.96 (1.52–2.46)
Others/no religion	1.09 (0.81–1.44)	1.18 (0.95–1.45)	1.37 (0.75–2.26)	1.20 (0.66–1.95)
**Household Wealth index**				
Poorest	2.95 (1.90–3.43)	2.39 (2.00–2.92)	1.21 (0.61–2.18)	1.40 (0.88–2.06)
Poorer	2.47 (1.59–3.72)	1.95 (1.63–2.39)	1.42 (0.84–2.26)	1.77 (1.23–2.44)
Middle	2.10 (1.37–3.16)	1.67 (1.39–2.06)	1.60 (1.04–2.38)	1.20 (0.87–1.60)
Richer	1.67 (1.08–2.47)	1.46 (1.20–1.81)	1.39 (1.01–1.95)	1.19 (0.93–1.49)
Richest (reference)	1	1	1	1
**Contextual-level factors**				
EAG-Assam versus other states	1.33 (0.70–2.26)	1.33 (0.79–1.77)	1.53 (0.51–3.97)	1.54 (0.61–2.85)
Community Illiterate women	1.00 (0.99–1.01)	1.00 (0.99–1.01)	0.99 (0.99–1.00)	0.99 (0.99–1.00)
Community poverty	0.88 (0.57–1.31)	0.87 (0.63–1.15)	1.05 (0.36–2.45)	0.54 (0.22–1.31)
**Variance (SE)**				
Community	1.62 (0.14)	1.02 (0.07)	0.0007 (0.0002)	0.79 (0.14)
District	0.84 (0.09)	0.40 (0.04)	0.69 (0.17)	0.32 (0.09)
State	1.4 90.39)	0.96 (0.32)	0.82 (0.41)	0.62 (0.29)
**MOR**				
Community	3.37 (3.07–3.81)	2.62 (2.46–2.79)	1.02 (NA)	2.12 (1.58–3.03)
District	2.40 (1.19–2.64)	1.82 (1.71–1.95)	2.21 (1.82–2.68)	1.72 (1.50–1.99)
State	2.77 (2.06–3.98)	2.54 (1.96–3.50)	2.38 (1.68–3.62)	2.33 (2.01–2.66)

**MOR:** Median odds ratio; CUV: received none of the eight basic vaccine doses; No-RI: received none of the five recommended vaccine doses delivered exclusively through the routine immunisation system; 95% CrI: Credible interval.

Random effects measures are also presented for all adjusted analyses (Tables 6, 8 and 10). In Table 6, results showed a significant variation of CUV across the communities (u-_CUV = _2.04, *p*<0.0001), districts (v-_CUV_ = 1.15, *p*<0.0001) and states (f-_CUV_ = 1.93, *p* = .001). These findings point to significant heterogeneity at each level as confirmed by corresponding MORs. To assess further the influence of compositional and contextual variables on random effects, we compared the null model (Model 1) with the three other models. By controlling for all compositional factors, the proportion of total variation attributed to community, district and state declined. Nonetheless, variation at these three levels remained highly significant (*p*<0.001), indicating that compositional characteristics may explain only a part of geographic variation. Consistently, MORs confirmed such heterogeneity.

After controlling for defined contextual variables in Model 3, community and district levels variances barely changed in comparison to Model 1 (Tables 6 & 8). Controlling for all compositional variables and contextual variables in Model 4 induced a substantial variances decrease in comparison to Model 1. Strikingly, the procedure showed little effect on proportional change of variance relative to Model 3. Correspondingly, clustering of on receipt of recommended vaccines remained significant at the community-, district- and state-levels, as indicated by respective MOR for CUV [3.44, (95% CrI: 3.14–3.79), 2.39 (95% CrI: 2.17–2.64) and 2.72 (95% CrI: 2.04–3.44)]. In stratified analyses by area of residence (Tables 9 & 10) controlling for all individual and contextual variables, significant variation of the outcome still persisted at community, district and states levels. This feature suggests that models did not fully explain contextual variation of non-vaccination. Furthermore, the level of community was more important in the determination of CUV status in rural settings while the state level was the most prominent in urban areas.

## Discussion

This is the first study to analyse unvaccinated children in India in order to appreciate potentially different contextual and compositional determinants associated with different vaccine delivery modes. Using multilevel modelling and the most recent representative probability sample of 65 617 children aged 12 to 23 months recruited from 34 states or territories of India, we found that 4.8% (95% CrI: 4.6–5.1) of Indian children were left out of India’s Universal Immunization Programme and had not received even a single vaccine dose (CUV). Results also showed wide inter-state variation ranging from 0% (Goa and Lakshadweep) to 20.9% (Tripura). In 2014, the World Health Organization certified India polio-free. India’s high-performing polio program will hence downscale efforts in future, and routine immunization services will shoulder the task of reaching every child. By documenting the magnitude of the coverage gap associated with failure to receive routine immunization and associated risk factors, our analysis provides key information to inform service delivery improvements in India. A much higher proportion of children had received no vaccinations from routine services, an indication of weak health systems. The prevalence of No-RI is 12.01% (95% CI: 11.68–12.35%) nationally, ranging from 0% (Lakshadweep) to 25.4% (Tripura). The prevalence of No-RI was above 20% in Tripura, Uttar Pradesh, Meghalaya and Arunachal Pradesh. CUV and no-RI children are concentrated in particular states, increasing the risk of transmitting VPDs to other unvaccinated and undervaccinated children.

Equity in coverage of maternal and child lifesaving interventions such as vaccinations in resource-limited countries remains a major focus of global health agenda [34], [35]. Subsequently, reaching unreached children is recognised as central to this vision [18]. Actually, those left out from vaccinations are thought to be at highest risk of VPDs and are also unlikely to access other essential child health services [36], [37]. Our multilevel analysis identifies key findings that are relevant to understanding the role of certain contextual and compositional factors in influencing non-receipt of any basic vaccine-dose in India, a country concentrating 25% of unvaccinated children globally [6].

Non-vaccination is highly associated with the mother’s and her partner’s educational attainments. Mothers that not reached at least 6 years of schooling had a higher risk to have unvaccinated children. Importantly, the effect of maternal schooling attainment appears less apparent for completely unvaccinated children in rural areas while paternal education appears systematically manifest for both outcomes in all settings. The finding relative to maternal education is consistent with a body of evidence from India and other resources limited settings. Thus, they provide further evidence that mother education remains a strong determinant of child vaccination in certain circumstances [13], [38]–[40]. The finding that husband’ education is associated with childhood vaccination is in line with previous reports [41]. Indeed, the protective role of husband’s education has been recognized [42], [43] as reinforcing factor for mothers propensity to seek for child’s vaccination. Finally, the positive association between parental education and non-vaccination lends credence to the importance of reinforcing demand-related factors.

Of note, three other individual-level effects are notably important. In unadjusted and multivariate models, the risk of being unvaccinated is strongly associated with all the compositional antenatal and postnatal covariates related to health services utilization by mothers. Thus, we found that children born from a mother who had not received tetanus toxin injection during pregnancy had a 4-fold risk of being unvaccinated. Furthermore, unvaccinated children were more likely to have mother who neither attended recommended full ANC visits nor PNC visit within the two weeks following birth. These associations remain significant even after controlling for all individual and contextual variables. These findings highlight the fact that the continuum of care throughout pregnancy and postpartum period is critically important for children vaccination in India. Indeed, the association between child vaccination and prenatal and postnatal care utilisation suggests that this pattern may be indicative of health services attendance during early childhood, as previously reported by Kogan [44]. Moreover, these findings are consistent with those of many others in resources limited settings [45], [46], though Choi & Lee found only such link for the subgroup of rural boys [47].

Socioeconomic factors are consistently linked to non-vaccination [48]–[50]. Accordingly, we showed that household wealth index was inversely associated with non-vaccination through a dose-response pattern while controlling for compositional and contextual variables. Importantly, this association appears mostly marked in rural settings.

Interestingly, our investigation found that the determinants of non-vaccination were similar between CUV and No-RI, but the magnitude of associations for individual factors such as maternal education, tetanus immunization during the pregnancy, ANC visits and child gender were usually stronger among No-RI. In order words, No-RI outcome usually accentuated the force of these associations. This pattern suggests that the polio programme operating largely in campaign mode in parallel to routine vaccination services seems to better reach undeserved groups such as very illiterate parents. Lessons from the polio programme’s approach to reaching underserved population groups may be useful for improving routine immunization services.

Turning to contextual variables, only place of residence was found to be significantly associated to non-vaccination. Meanwhile, residing in a rural area was associated with non-vaccination in unadjusted and multivariate models when considering only contextual factors. Conversely, this variable was found protective when taking into account both compositional and contextual factors. This inverse association between place of residence and non-vaccination contradicts the impressive body of evidence reported from both Asia and Africa [49]–[51]. Nonetheless, other African studies showed that children living in urban places are more likely to be non-vaccinated compared to their rural counterparts [42], [52]. This apparent discrepancy raises the question of whether the so called “urban advantage” remains pertinent in regard to growing urban population and for whom access to health services may be precarious [11], [53], [54].

Overall, even after controlling for observed characteristics, unexplained heterogeneity in non-receipt of any vaccine remains significant at contextual levels net of what could be attributed to compositional factors. In terms of the relative importance of the three contextual levels, the community-level was observed to be relatively more important in rural settings while the state-level appeared more influential in urban areas in the determination of non-receipt of any vaccine. Finally, none of the considered contextual variables seemed to have a greater impact on non-vaccination relative to compositional characteristics. Therefore, the relationship between the non-vaccination and the context may be more complex than captured in this study. The evidence of a state-level clustering effect, as well as the clustering of district and community levels, suggests that unexplained factors should be sought in future analysis. In particular, we could not assess the potential contribution of the availability of health facilities (only available for rural areas in the DHLS-3), state or district level governance.

Our study has several strengths. Firstly, it is original as we used more stringent measures of non-vaccination compared to traditional measure of non-vaccination based only on coverage of DPT1 [6]; second, we modelled the non-receipt of vaccines throughout two different delivery systems; third, we used highly computational but robust statistical techniques within a multilevel framework. Finally, we minimized potential selection bias and achieved nationwide representative estimates (generalization) by using India’s most recent publicly available nationally representative survey data, the DLHS-3.

There are some limitations to this study. The cross-sectional nature of the data limits the ability to draw causal inferences. We also recognized that our study may be potentially limited by the fact that the determination of vaccination status was based mainly on mother or care giver report, which may be less precise than information provided by health card. Nevertheless, this practice is commonly used by the Demographic and Health Surveys (DHS) which form the basis for the DLHS since its first series [55]. Moreover, other studies have shown that mothers’ reports of their children’s vaccination status are fairly accurate [56]–[58]. Finally, additional information on unmeasured individual and community level variables by data source such as health services characteristics that were only available for rural areas, and cultural norms and beliefs, difficult to collect in such large-scale survey, would have also benefited this study.

Our results have potentially important implications for public health policies and programs aimed at reducing non-vaccination in India. At the individual level, the results suggest that health programs need to be adapted geographically and to focus on attracting poor children particularly in rural settings and less educated women and encouraging them to use health services including vaccination. Our findings further reiterate the urgent need for a comprehensive maternal health package that addresses the spectrum of maternal and extended newborn care –envisaged as critical components in achieving targets 4, 5a and 5b of the UN Millennium Development Goals [59]. The persistence of significant community-, district- and state-level variation in non-vaccination illustrates that current large population surveys such as DLHS are insufficient in measuring the range of cultural influences on health-seeking behavior, and more focused-research is needed to understand the dynamics of contextual influences on individuals.
